# Trends in the use of oral contraceptives among adolescents and young women in Spain

**DOI:** 10.1186/s12978-016-0239-4

**Published:** 2016-09-23

**Authors:** Pilar Carrasco-Garrido, Ana López de Andrés, Valentín Hernández-Barrera, Isabel Jiménez-Trujillo, Mercedes Esteban-Peña, Napoleón Pérez-Farinós, Rodrigo Jiménez-García

**Affiliations:** 1 Preventive Medicine and Public Health Teaching and Research Unit, Health Sciences Faculty, Rey Juan Carlos University, Avda. Atenas s/n. 28922-Alcorcón, Madrid, Spain; 2 Training and Research Unit Health Madrid, City of Madrid, Spain, 28009 Madrid, Spain; 3 Food Safety Agency, Ministry of Health, 28071 Madrid, Spain

## Abstract

**Background:**

We aimed to determine the prevalence of consumption of oral contraceptives (OCs) among adolescents and young women living in Spain and to identify the factors associated with this consumption.

**Methods:**

We performed a cross-sectional study on the consumption of OCs by women aged 15–30 years residing in Spain. We used secondary individualized data from the 2006 (*n* = 2513) and 2012 (*n* = 1530) Spanish National Health Surveys. The dependent variable was the use of OCs in the previous 2 weeks. Independent variables included sociodemographic characteristics, comorbidity, lifestyle, and healthcare resource utilization.

The prevalence of OC consumption was analysed by investigating the changes observed between 2006 and 2012. We used multivariate logistic regression to identify the independent factors associated with OC use in each year.

**Results:**

In 2006, 14.42 % of women reported using OCs; this percentage dropped to 10.21 % in 2012 (*p* < 0.05). Multivariate analysis revealed an association between OC use and visits to the gynaecologist (AOR, 5.60 [95 % CI, 2.93–10.73] in 2006; and AOR, 3.55 [95 % CI, 1.30–9.73] in 2012), Pap smear tests (AOR, 1.8 [95 % CI, 1.23–2.87] in 2006; and AOR, 2.42 [95 % CI, 1.30–4.51] in 2012), and smoking in 2006 (AOR, 1.42 [95 % CI, 1.04–1.93]).

**Conclusions:**

There was a significant decrease in OC use from 2006 to 2012 among adolescents and young women living in Spain. In the present study, consumers of OCs were women who visited a gynaecologist more often and complied more with preventive measures such as Pap smear testing. Also, women who reported having used OCs were more likely to smoke than the rest of the study population, although the smoking habit is a risk factor for thrombotic events in women who take OCs.

## Plain english summary

The introduction of the oral contraceptive pill has allowed women to exercise greater control over the childbearing phase of their life. The objectives of the present study are to describe, using the data drawn from the 2006 and 2012 Spanish National Health Surveys (SNHS), the prevalence of contraceptive methods and identify factors associated with the use of contraception by adolescents and young women resident in Spain. Our study population comprised women aged 15 to 30. A total of 4043 women were included: 2513 from the 2006 SNHS and 1530 from the 2012 SNHS. The interviews were conducted between June 2006 and June 2007 (SNHS2006); and from July 2011 to June 2012 (SNHS2012). The surveys use multistage cluster sampling, with proportional random selection of primary and secondary sampling units (towns and sections, respectively), and the final units (individuals) are selected by means of random routes and sex- and age-based quotas.

This study found a significant decrease in OC use among adolescents and young women living in Spain from 2006 to 2012. The decrease in consumption of OCs in Spain has two possible explanations: first, the decrease could be due to a change in the social perception of oral contraceptives in Spain in terms of family planning policy, because the male condom is the first choice, and second, the economic recession seems to have affected some indicators of sexual and reproductive health. The OC consumers in our study were women who visited a gynaecologist more often and complied more with preventive measures such as Pap smear testing. Also, women who reported having used OCs were more likely to smoke than the rest of the study population, although the smoking habit is a risk factor for thrombotic events in women who take OCs.

## Article summary

### Strengths

- The strengths of our study include its large population-based sample size, a randomly selected population, the use of a standardized survey, training of the data collectors, and our ability to control for a broad range of major covariates.

### Limitation

- We used data from a cross-sectional survey, we cannot determine cause and effect relationships between the variables associated with OC use.
- The SNHSs, rather than identifying specific active pharmaceutical ingredients, identify groups of medicines for specific diseases or disorders. Consequently, we do not know whether OCs are COCs or whether they are progestin-only pills (POPs, the Mini-Pill).

## Background

Modern contraception is one of the great public health achievements of the last century. The introduction of the oral contraceptive (OC) pill has allowed women to exercise greater control over the childbearing phase of their life. Several studies show that OCs are the most popular form of reversible contraception in developed countries [1–4]. However, many teenage pregnancies are unplanned and generate a significant societal cost and potential individual risk [5]. Around the world, approximately 16 million girls aged 15 to 19 and some one million girls under 15 give birth every year, and 85 % of these pregnancies are unplanned [6]. Every year in Spain, there are about 4000 term pregnancies in adolescents under the age of 17 [7]. Investigations carried out on the use of contraception among Spanish women show that the male condom is the most common method, followed by OCs [8, 9]. OCs in Spain are available with prescription or as over-the-counter (OTC) products. Oral contraceptives are available to Spanish women age 15 and older at pharmacies, and there is no age limit on the availability of emergency contraception. Contraceptives are reasonably priced and are partially subsidized. The price has not changed in recent years.

These findings are reflected in the Sexual Attitude of Females in Europe Survey, which reveals significant differences between the low use of pills in Spain and the high demand in other countries (Italy, UK, France, Germany, Sweden, Denmark, Norway, the Czech Republic, Austria, Estonia, Latvia, Lithuania, and the Russian Federation) [5, 10]. The Health Behaviour in School-Aged Children Study 2009/2010 showed that, among 15-year-old Spanish adolescents, 8 % of girls and 3 % of the respondents’ male partners reported using contraceptive pills during their last intercourse [11]. However, forthcoming social and legislative changes in the area of sexual and reproductive health in Spain could have an impact on the patterns of use of contraception among adolescents and young women. An example of a policy change, that has already been implemented, is the OTC availability of the emergency contraception in Spanish pharmacies since 2010.

In this context, we aimed to determine the prevalence of OC consumption among adolescents and young women living in Spain and to identify the factors associated with this consumption. We also analysed how this prevalence changed from 2006 to 2012.

## Methods

We conducted a nationwide, descriptive, cross-sectional epidemiological study on the consumption of OCs by adolescents and young women aged 15–30 years residing in Spain. We used individualized secondary data from the 2006 and 2012 Spanish National Health Surveys (SNHS) [12, 13]. SNHSs are interview-based surveys conducted by the Ministry of Health in collaboration with the National Statistics Institute (Instituto Nacional de Estadística) that target a large sample of the non-institutionalized Spanish population. The surveys use multistage cluster sampling, with proportional random selection of primary and secondary sampling units (towns and sections, respectively). The final units (individuals) are selected by means of random routes and sex- and age-based quotas. The 2006 survey included 29,478 adults of both sexes interviewed between June 2006 and June 2007; the 2012 survey included 20,007 persons interviewed from July 2011 to June 2012. Details of SNHS methodology are described elsewhere [12, 13]. The variables included in our study were created on the basis of a series of identically worded questions asked in the two surveys used. Information was collected by direct home-based computer-assisted interviews. The persons interviewed responded to the questionnaires. Surveyors were previously trained in basic communication skills, related procedures and specific training on the questionnaire to be used.

Our study population comprised women aged 15 to 30. A total of 4043 women were included: 2513 from the 2006 SNHS and 1530 from the 2012 SNHS.

The information to create the dependent variables was obtained from the answers “yes” or “no” to the question “*In the last two weeks have you taken the contraceptive pill*?”. Respondents who answered “yes” were then asked the question *“Were they prescribed for you by a doctor?”* Those who answered yes to the first question, regardless of the answer to the second one, were considered consumers of OCs.

The independent variables were the primary sociodemographic characteristics of the population, namely, age, nationality, marital status, occupational status, educational level, total household monthly income in euros, and social class. The categories for these variables are shown in Table 1.

**Table 1:** Sociodemographic characteristic of the study population. Spanish National Health Surveys 2006 and 2012

		SNHS 2006 N (%)	SNHS 2012 N (%)
Age	15–22	827 (32.91)	612 (40)
	23–30	1686 (67.09)	918 (60)
Nationality	Immigrants	394 (15.68)	205 (13.40)
	Spanish	2119 (84.32)	1325 (86.60)
Marital status	Not married	1821 (72.46)	1278 (83.58)
	Married	692 (27.54)	251 (16.42)
Occupational status	Employed	1355 (53.92)	605 (39.54)
	Unemployed	313 (12.46)	264 (17.25)
	Inactive	845 (33.63)	661 (43.20)
Educational level	Junior school	382 (15.56)	669 (43.72)
	High school	1523 (62.04)	567 (37.06)
	University and higher education	550 (22.4)	294 (19.22)
Monthly income	≤1200 euros	842 (33.77)	536 (35.61)
	> 1200 euros	1651 (66.23)	969 (64.39)
Social class	High	317 (16.52)	249 (16.93)
	Middle	667 (34.76)	472 (32.09)
	Low	935 (48.72)	750 (50.99)

Self-rated health was analysed as a dichotomous variable (very good/good vs. fair/poor/very poor).

Variables related to comorbid chronic illness were also analysed. These were collected using the question “*Has the doctor told you that you suffer from one of these diseases?”* The list of diseases comprised arterial hypertension, hypercholesterolemia, heart disease, and diabetes. Women who reported suffering from one or more of these conditions were classified as “chronic sufferers”. The presence of depression or anxiety (psychological disorders) was assessed using the following questions: 1) *“Have you suffered from depression or anxiety in the last 12 months?”* And for those who answered “yes” 2) *“Has your doctor confirmed the diagnosis?”* Only women who answered “yes” to both questions were considered to have a psychological disorder.

The lifestyle-related variables used in the study were as follows: currently smoking (yes/no), consumption of alcoholic beverages in the 2 weeks prior to the survey (yes/no), leisure time physical activity (intense or moderate vs. none), and self-reported body mass index (calculated from self-reported height and weight) categorized as <25, 25–30 and >30.

In order to assess the use of healthcare resources, subjects were asked about the following: i) visits to their general practitioner in the last 12 months, ii) type of health insurance (public vs. private), iii) visits to the gynaecologist in the last 12 months, iv) Pap smear test in the last 12 months, v) use of family planning services, vi) consumption of any type of medication in the last two weeks, and vii) use of any alternative medicine in the last two weeks.

### Statistical analysis

We calculated the prevalence of total consumption of OCs for each of the two surveys and according to the study variables. For the bivariate comparison of proportions, Pearson’s *χ*2 or Fisher’s exact test was applied, with values of *p* < 0.05 taken as significant. To estimate the independent effect of OC use on study variables, we also obtained the corresponding adjusted odds ratio (AOR) through multivariate logistic regression analysis. All variables that showed a significant association (*p* < 0.05) in the bivariate analysis were included in the multivariate analysis, along with variables that were considered relevant in the scientific literature.

Finally, to assess the time trend in the consumption of OCs during the study period, we combined the respective databases of the two SNHS and calculated the crude and adjusted odds ratios for having consumed OCs in 2012 compared with 2006. To calculate the AOR, all variables that proved to be predictive in either SNHS were included in the multivariate logistic regression.

Estimates were made using the *svy* (survey commands) functions of the Stata program (STATA Corp, College Station, Texas, USA), which enabled us to incorporate the sampling design and weights into all of the statistical calculations (descriptive, *χ*^2^, logistic regression). Statistical significance was established as a two-tailed α < 0.05.

## Results

The study was based on data from 4043 women (2513 from the 2006 SNHS and 1530 from the 2012 SNHS) aged 15–30 residing in Spain who answered the question on consumption of OCs in the two weeks immediately preceding the survey. Descriptions of the sociodemographic characteristics of the study population are shown in Table 1.

The 2006 SNHS data revealed that 14.42 % (95 % CI, 12.75–16.27) of adolescents and young women residing in Spain reported having taken the contraceptive pill in the two weeks leading up to the survey. This proportion decreased to 10.21 % (95 % CI, 8.61–12.07) in 2012, and the crude difference proved statistically significant association (*p* < 0.05). Consumption was almost entirely by prescription, with the percentages of self-medication being only 5.90 % (95 % CI, 3.26–10.44) in 2006 and 4.84 % (95 % CI, 2.44–9.35) in 2012.

Table 2 shows data on the prevalence of consumption of OCs according to the sociodemographic characteristics of the population and lifestyle variables. The bivariate analysis revealed that the prevalence of OC use was highest in women aged 23 to 30 years during the study period (17.375 in 2006 and 13.32 % in 2012), *p* < 0.05. It is noteworthy that the frequency of OC consumption was higher for university-educated women, women with higher incomes, and smokers, a finding that was repeated to a significant degree in both surveys (*p* < 0.05). Identical results were obtained for alcohol consumption.

**Table 2:** Prevalence of oral contraceptive use in adolescents and young women living in Spain, according to socio-demographic variables and lifestyle. Spanish National Health Surveys 2006 and 2012

		Oral contraceptive use	
		SNHS 2006 % (95 % CI)	SNHS 2012 % (95 % CI)
Age*†	15–22	9.71 (7.47–12.52)	5.93 (4.33–8.07)
	23–30	17.37 (15.12–19.87)	13.32 (10.91–16.17)
Nationality	Immigrants	14.63 (10.58–19.87)	8.96 (5.21–14.96)
	Spanish	14.37 (12.60–16.35)	10.52 (8.83–12.47)
Marital status	Not married	13.77 (11.90–15.89)	9.87 (8.24–11.79)
	Married	16.61 (13.19–20.71)	12.03 (7.52–18.71)
Occupational status*†	Employed	18.45 (15.84–21.37)	13.28 (10.53–16.62)
	Unemployed	11.91 (8.37–16.67)	9.22 (6.16–13.58)
	Inactive	9.70 (7.53–12.42)	8.08 (5.91–10.94)
Educational level*†	Junior school	11.00 (7.75–15.38)	7.69 (5.55–10.57)
	High school	14.09 (12.00–16.47)	10.83 (8.41–13.82)
	University and higher education	18.16 (14.35–22.72)	15.08 (10.93–20.44)
Monthly income†	≤ 1200 euros	12.89 (10.09–16.34)	7.75 (5.64–10.57)
	> 1200 euros	15.36 (13.32–17.66)	11.34 (9.25–13.83)
Social Class	High	19.14 (13.82–25.67)	11.40 (7.76–16.45)
	Middle	15.72 (12.29–18.77)	11.70 (8.90–15.23)
	Low	14.03 (11.11–17.45)	8.87 (6.74–11.57)
Smoking habit*†	Non smoker	12.04 (10.21–14.15)	8.98 (7.19–11.15)
	Smoker	19.11 (15.89–22.81)	13.24 (10.15–17.11)
Alcohol consumption*	No	11.38 (8.86–14.49)	9.90 (8.12–12.03)
	Yes	15.92 (13.82–18.28)	11.37 (8.14–15.68)
Physical activity	Moderate	15.33 (13.08–17.89)	11.10 (8.90–13.76)
	inactive	13.30 (10.92–16.11)	9.87 (7.56–12.80)
Body Mass Index Kg/sq.m	< 25	14.19 (12.28–16.35)	11.31 (9.35–13.63)
	25–29	16.91 (12.72–22.14)	6.52 (3.96–10.58)
	30 or over	16.30 (9.02–27.65)	9.18 (4.15–19.11)
Total consumption of OCs		14.42 (12.75–16.27)	10.21 (8.61–12.07)

*Statistically significant association of consumption in SNHS 2006 (*p* < 0.05)

†Statistically significant association of consumption in SNHS 2012 (*p* < 0.05)

Table 3 shows the consumption of OCs as reported by study participants and according to comorbid conditions and use of healthcare resources. In 2006, OC consumption was greater among adolescents and young women with psychological disorders (19.16 %, *p* < 0.05). Both surveys also showed that consumption was more common among those who had made at least one visit to a gynecologist in the past year than among those who had not. In addition, the Pap smear test was more prevalent among women using the pill (28.42 % vs. 7.19 % in 2006 and 15.96 % vs. 3.69 % in 2012) *p* < 0.05 and women using family planning services.

**Table 3:** Prevalence of oral contraceptive use in adolescents and young women living in Spain, according to comorbidity and use of healthcare resources. Spanish National Health Surveys 2006 and 2012

		Oral contraceptive use	
		SNHS 2006 % (95 % CI)	SNHS 2012 % (95 % CI)
Self-assessment of health status	Very good / Good	13.75 (11.91–15.82)	10.14 (8.45–12.12)
	Fair / Poor / Very poor	17.30 (13.58–21.78)	10.81 (6.65–17.10)
Chronic illness*	No	13.56 (11.77–15.46)	9.76 (8.08–11.74)
	Yes	19.78 (14.96–25.68)	13.47 (9.02–19.63)
Psychological disorders*	No	14.03 (12.31–15.96)	10.04 (8.40–11.97)
	Yes	19.16 (13.33–26.76)	13.25 (7.45–22.46)
Medical consultation*	No	12.40 (10.59–14.47)	9.75 (7.86–12.05)
	Yes	18.48 (15.20–22.29)	11.25 (8.52–14.70)
Type of health insurance coverage	Private	14.78 (8.39–24.72)	14.12 (9.71–20.08)
	Public	14.41 (12.69–16.31)	9.61 (7.94–11.60)
Gynecologist visits in preceding 12 months*†	No	2.27 (1.37–3.74)	1.95 (0.9–4.17)
	Yes	20.09 (17.76–22.64)	13.86 (11.66–16.40)
Pap smear in preceding 12 months*†	No	7.19 (5.43–9.48)	3.69 (2.38–5.68)
	Yes	22.48 (19.74–25.49)	15.96 (13.32–19.00)
Family planning services in preceding 12 months*†	No	12.85 (11.25–14.64)	9.81 (8.23–11.67)
	Yes	41.78 (31.12–53.27)	19.39 (10.05–34.11)
Medication consumption*†	No	14.24 (12.49–16.18)	9.46 (7.90–11.28)
	Yes	16.46 (11.30–23.35)	19.93 (12.01–31.23)
Alternative medicines*†	No	14.11 (12.42–15.99)	9.66 (8.07–11.53)
	Yes	23.09 (14.32–35.02)	28.39 (16.44–44.40)

*Statistically significant association of consumption in SNHS 2006 (*p* < 0.05)

†Statistically significant association of consumption in SNHS 2012 (*p* < 0.05)

The multivariate logistic regression analysis showed the effect of each of the study variables, duly adjusted for the remainder, on the overall consumption of OCs in the study sample. These estimates were made for both surveys and are shown in Table 4.

**Table 4:** Factors associated with consumption of oral contraceptives in adolescents and young women living in Spain. Spanish National Health Surveys 2006–2012

Variables		SNHS 2006			SNHS 2012		
		Adjusted OR	95 % CI	*p*-value	Adjusted OR	95 % CI	*p*-value
Age	15–22	1			1		
	23–30	0.92	(0.61–1.36)	0.867	1.29	(0.80–2.05)	0.257
Smoking habit	Non smoker	1			1		
	Smoker	1.42	(1.04–1.93)	0.020	NS		0.547
Gynecologist visits in preceding 12 month	No	1			1		
	Yes	5.60	(2.93–10.73)	0.000	3.55	(1.30–9.73)	0.022
Pap smear test in preceding 12 months	No	1			1		
	Yes	1.87	(1.23–2.87)	0.003	2.42	(1.30–4.51)	0.004
Family planning services in preceding 12 months	No	1			1		
	Yes	3.56	(2.14–5.93)	0.000	NS		0.147
Alternative medicines	No	1			1		
	Yes	NS		0.851	2.83	(1.32–6.04)	0.008

Data are expressed as adjusted odds ratio (AOR) and 95 % confidence intervals (95 % CI)

*NS* non-significant association

In 2006, the variables that were independently and significantly associated with a higher likelihood of OC use were smoking (AOR 1.42; 95%CI 1.04–1.93), *p* = 0.020, visits to the gynaecologist in the preceding 12 months (AOR, 5.6; 95 % CI, 2.93–10.73), *p* = 0.000, having a Pap smear test (AOR 1.85; 95%CI, 1.23–2.87), *p* = 0.003, and visiting family planning services (AOR, 3.56; 95 % CI, 2.14–5.93), *p* = 0.000.

In 2012, some of the predictive variables were the same as in 2006, such as visits to the gynaecologist (AOR 3.55; 95 % CI, 1.30–9.73), *p* = 0.022, Pap smear tests (AOR 2.42; 95 % CI 1.30–4.51), *p* = 0.004, although a new variable, use of alternative medicines (AOR, 2.83; 95 % CI, 1.32–6.04), *p* = 0.008, was also assessed.

The adjusted OR for having consumed OCs in 2012 with respect to 2006 was 0.67 (95 % CI, 0.52–0.85), *p* = 0000. Therefore, after controlling for possible confounders, the likelihood of having consumed OCs in 2012 was lower than it had been 6 years previously.

## Discussion

National health surveys have become a valid and widely used research tool for ascertaining patterns of OC use in the female population [14–17]. According to data from a recent report with updated information on the sexual behaviour of adolescents and the use of contraception, the hormonal contraceptive method most commonly used by adolescents is the combined oral contraceptive (COC) (55.6 % of young women have used an OC) [18]. Recent studies on the development of OCs over time show that this method has become increasingly common among Spanish women [15, 19, 20]. Use of hormonal contraception is mainly among women aged 20 to 30 [5, 9].

However, the present study revealed a significant decrease in the consumption of OCs during the study period 2006–2012 (AOR, 0.67; 95 % CI, 0.52–0.85), which is consistent with the results of a recent study by Zelić-Kerep et al. [21], who quantified and analysed trends in the use of oral hormonal contraceptives in the city of Zagreb, Croatia. Their results showed a 76 % decrease in the use of hormonal contraceptives from 2008 to 2009.

In the 2012, the prevalence of OC use reached 10.21 % among adolescents and young women living in Spain. This percentage was considerably lower than the 16.2 % found by Cea-Soriano et al. [22] in a study to determine prescription contraceptive use in the United Kingdom with data from The Health Improvement Network (THIN). Løkkegaard [23] also showed higher percentages of OC use among Danish female adolescents. By age 17, more than 50 % of Danish female adolescents had had a prescription for hormonal contraceptives. Similarly, a recent study showed that, among Portuguese adolescents, 33 % of girls and 18 % of the respondents’ male partners had used contraceptive pills during their most recent intercourse [11].

The decrease in consumption of OCs in Spain has two possible explanations. First, different investigations conducted in Spain on the use of contraceptive methods among the female Spanish population indicate that condoms are the method most used in our country. The rate of condom use in Spain is relatively high (71 %) in adolescents and young adults [8] in comparison with other European countries, such as Greece (53.87 %) [24] and Sweden (68 %) [25]. Many investigations highlight the fact that the use of condoms in the Spanish population has not decreased; on the contrary, it continues to increase in percentage terms (38.8 % in 2007 to 46.7 % in 2012). Second, the economic recession seems to have affected some indicators of sexual and reproductive health. Since the beginning of the recession, contraceptive use has declined [26], and access to contraception has become difficult because of cost [27]. In this sense, if our National Health system continues to stop funding new-generation OCs, which are more efficient and safer, new barriers to their distribution would appear [28]. Therefore, young Spanish women would choose other contraceptive methods. In addition, since 2010, the emergency contraception has been sold directly in Spanish pharmacies without the need for a medical prescription. It is possible that when women were provided EC without a prescription, they were more likely to use the method [29, 30].

The association between copayment and hormonal contraception was analysed by Kazerooni et al. [31] and Poncet et al. [32], whose results show the relationship between socioeconomic status and the use and method of contraception among women residents in France.

OCs remain an effective form of contraception, despite the availability of newer methods such as intrauterine devices or the extended-cycle pill. Generic and OTC contraceptives are favored by third-party payers. As a result, potentially exciting developments in the safety of branded oral contraceptives may have gone unnoticed [33]. However, Foster et al. [34], showed that the interest in OTC access to oral contraceptives is high. If out-of-pocket costs for such pills are low, OTC access could have a significant effect on the use of effective contraceptives and unintended pregnancy. In this sense, cost will continue to determine whether these new contraceptive methods are accepted, unless substantial health benefits can be adequately proven. The economic recession may have played a role in this decrease.

A lifestyle analysis revealed that, in 2006, women who reported having used OCs were more likely to smoke than the rest of the study population (AOR, 1.42; 95 % CI, 1.04–1.93). Several studies have reported that OCs and smoking-derived nicotine are known to synergistically increase the risk and severity of cerebral ischemia in women [35]. COC users who are current smokers have a tenfold higher risk of myocardial infarction and an almost threefold risk of stroke than those who do not [36]. The study by Berlín et al. in France [37] showed that hormonal contraception in adolescent girls can accelerate the metabolism of cotinine. The increased rates of smoking among teenagers are not unique of Spain. Adolescents start smoking in response to social influences, emulating the behavior of friends and family members, and are influenced by the media. There is a possible relation between smoking and early sexual initiation. Recent studies show that the smoking prevalence is higher among disadvantaged groups [38], and also that girls with low educational and socioeconomic status tend to experience sex at a younger age. This situation is a window of opportunity to promote hormonal contraception [39]. Interventions targeting young women should be developed to increase their knowledge about contraceptive methods and the risk of smoking.

As for preventive measures, our results show that during the study period, OC users were more likely to have a Pap smear test than women who did not use OCs. We must also bear in mind that the International Agency for Research on Cancer (IARC) has classified COCs as carcinogenic for humans. Therefore, regular smear tests in sexually active women can be considered the best method for the prevention of cervical cancer.

As for healthcare resource–related variables, our results revealed that the variable with the greatest strength of association with OC use in both surveys was visits to the gynaecologist in the previous 12 months (AOR, 5.60 [95 % CI, 2.93–10.73] in 2006 and AOR, 3.55 [95 % CI, 1.30–9.73] in 2012). Adolescents are a special group who need motivational counselling and care to ensure that they adhere to the daily routine and to continue regular use [40, 41]. As a result, gynaecologists attempt to adapt prescriptions to the specific characteristics of available pills in order to improve adherence [42]. A working knowledge of contraception will assist the paediatrician and gynaecologist in both promoting sexual health and treating common adolescent gynaecological problems.

Family planning services were also associated with OC use. In 2006, young women who reported having used OCs were more likely to visit family planning services than women who did not (AOR, 3.56 [95 % CI, 2.14–5.93]). These results are consistent with those of other studies on the utilization and perception of health services, such as the REPROSTAT project (Reproductive health indicators in the European Union), which highlighted that only 31 % of the youths interviewed had ever visited a health center to receive information on contraception, pregnancy, abortion, and STIs [43]. In the same line Darney et al. [44] in a study conducted in Mexico, to determine associations between age and patient-reported quality of family planning services among adolescents and young women, showed that although a lower proportion of adolescents reported long-acting reversible contraception (LARC) use, in comparison with young women, the adolescents using hormonal and LARC had significantly lower odds of reporting high-quality care compared with women aged 25–29. The role of family planning services is essential because the evidence supports that teenagers are more likely to choose a LARC method and continue with that method if they are offered [45].

Finally, in the 2012 SNHS, women who reported having used OCs were more likely to use alternative medicines than the rest of the study population (AOR, 2.83 [95 % CI, 1.32–6.04]). It is known that interactions between hormonal contraceptives and other herb-drugs can reduce the efficacy of OCs [46]. In the USA, Murphy et al. [47] evaluated the effect of the herbal remedy St. John’s Wort on OCs and found that treatment with St. John’s Wort was associated with a significant 13–15 % reduction in exposure to the OC.

The strengths of our study include its large population-based sample size, a randomly selected population, the use of a standardized survey, training of the data collectors, and our ability to control for a broad range of major covariates, including sociodemographic factors and variables associated with the use of health services. However, our study is also subject to a number of possible limitations. First, since we used data from a cross-sectional survey, we cannot determine cause and effect relationships between the variables associated with OC use. Second, the conclusions drawn are difficult to generalize, because the survey has not been validated for the consumption of medication. Third, information obtained from interviews is subjective and may be subject to recall errors or the tendency of subjects to give socially desirable responses. Another possible limitation of SNHSs is that, rather than identifying specific active pharmaceutical ingredients, they identify groups of medicines for specific diseases or disorders. Consequently, we do not know whether OCs are COCs or whether they are progestin-only pills (POPs, the Mini-Pill). Furthermore, the SNHS does not allow us to differentiate between women who took emergency contraceptives and women who had taken any kind of OC or other method used beside OCs. In the same line, the SNHS does not include data regarding LARCs use, among the population consulted, but other studies have found that the use of LARC is very low in Spain [48] compared with other countries, for example Mexico [44].

Finally, the SNHS does not include data about the sexual behaviour or reproductive and contraceptive knowledge of the population consulted. Finally, another limitation is that younger women who still live at their parents’ house because the they have not completed high school/university and/or have not started working full time, are affected by factors such as economic status and parental education.

Lastly, given that the initial response rate to the 2006 SNHS was 65 % and the initial response rate to the 2012 SNHS was 61 %, a possible non-response bias must be considered [12, 13]. According to the National Statistics Institute (INE), the initial non-response rate in both surveys was slightly higher among female, non-Spanish subjects, and those in the 40–65 age groups [49, 50].

## Conclusion

We observed a significant decrease in OC use among adolescents and young women living in Spain from 2006 to 2012.

In the present study, consumers of OCs were women who visited their gynaecologist often and followed preventive measures such as Pap smear testing. Also, women who reported having used OCs were more likely to smoke than the rest of the study population, although the smoking habit is a risk factor for thrombotic events in women who take OCs.
